# Synergy of SARS-CoV-2 and HIV-1 Infections in the Human Brain

**DOI:** 10.3390/pathogens15010089

**Published:** 2026-01-13

**Authors:** Rajnish S. Dave, Howard S. Fox

**Affiliations:** Department of Neurological Sciences, University of Nebraska Medical Center, Omaha, NE 68198-5930, USA

**Keywords:** SARS-CoV-2, COVID-19, brain, neuropathology, PWH, HIV-1, CNS

## Abstract

This review explores the interplay between SARS-CoV-2 and HIV-1 infections within the human brain, highlighting the significant neurological implications of these viral infections. SARS-CoV-2 can infect the central nervous system (CNS), with evidence of the virus detected in various brain regions, including the hypothalamus, cerebellum, and olfactory bulb. This infection is linked to microglial activation and neuroinflammation, which can lead to severe neurological outcomes in affected individuals. Autopsy studies revealed microglial changes, including downregulation of the P2RY12 receptor, indicating a shift from homeostatic to inflammatory phenotype. Similar changes in microglia are found in the brains of people with HIV-1 (PWH). In SARS-CoV-2, the correlation between inflammatory cytokines, such as IL-1, IL-6, and MCP-1, found in cerebrospinal fluid and brain tissues, indicates significant neurovascular inflammation. Astrogliosis and microglial nodules were observed, further emphasizing the inflammatory response triggered by the viral infections, again in parallel to those found in the brains of PWH. Epidemiologic data indicate that although SARS-CoV-2 infection rates in PWH mirror those in People without HIV (PWoH) populations, Long-COVID prevalence is markedly higher among PWH. Evidence of overlapping cognitive impairment, mental health burden, and persistent neuroinflammation highlights diagnostic complexity and therapeutic gaps. Despite plausible mechanistic synergy, direct neuropathological confirmation remains scarce, warranting longitudinal, biomarker-driven studies. Understanding these interactions is critical for developing targeted interventions to mitigate CNS injury and improve outcomes.

## 1. Introduction

Coronavirus Disease 2019 (COVID-19) is caused by the Severe Acute Respiratory Syndrome Coronavirus 2 (SARS-CoV-2), a highly contagious virus that affects the respiratory system [1,2]. The COVID-19 pandemic has resulted in a global health crisis, with one of the most concerning consequences being the virus’s immediate impact on lung function and its long-term effects on the Central Nervous System (CNS), manifested as “Long-COVID” [3,4]. COVID-19 frequently causes characteristic neurological symptoms, such as anosmia (loss of smell) and ageusia (loss of taste), in those who contract the virus [5].

The SARS-CoV-2 virus, a single-stranded RNA virus, belongs to the family of Coronaviruses [6]. Different coronaviruses are closely related and fall under the Betacoronavirus genus. SARS-CoV-2 virion includes four structural proteins: nucleocapsid, membrane protein, envelope, and spike. The spike S-glycoprotein (S protein) is inserted into the virion’s membrane in multiple copies, forming the characteristic crown-like appearance. Coronaviruses are known to cause respiratory tract infections in mammals and birds, with symptoms ranging from mild to severe and potentially lethal. Endemic coronaviruses, such as Human coronavirus OC43 (HCoV-OC43), Human coronavirus HKU1 (HCoV-HKU1), Human coronavirus 229E (HCoV-229E), and Human coronavirus NL63 (HCoV-NL63), typically result in mild upper-respiratory infection syndromes [7]. However, some cause only a mild common cold syndrome. In contrast, others, such as Severe Acute Respiratory Syndrome Coronavirus (SARS-CoV), Middle East Respiratory Syndrome Coronavirus (MERS-CoV), and SARS-CoV-2, can be highly lethal. The SARS-CoV epidemic was reported in 2002–2004, while Middle East respiratory syndrome (MERS) infection was first reported in Saudi Arabia in 2012, and SARS-CoV-2/COVID-19 in 2019 [8,9,10]. Horseshoe bats of the Rhinolophus genus are the long-term reservoirs of SARS-CoV and SARS-CoV-2 [6,11]. Dromedary camels are a reservoir host of the MERS-CoV [12].

**Figure 1:**
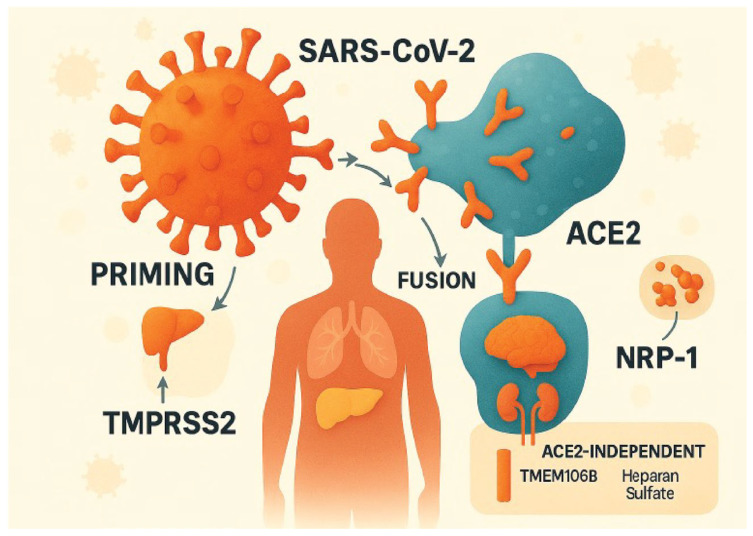
SARS-CoV-2 entry mechanisms in human cells. The canonical pathway involves the viral spike (S) protein binding to the ACE2 receptor, followed by priming by TMPRSS2 and subsequent fusion, which enables viral genome entry. Neuropilin-1 (NRP-1) acts as a co-factor enhancing infectivity. An alternative ACE2-independent route, observed in certain variants, utilizes TMEM106B and heparan sulfate for viral entry, as shown in the inset. Organs depicted reflect ACE2 expression patterns, including lungs, liver, kidneys, and brain.

SARS-CoV-2 enters human cells, utilizing the Angiotensin-Converting Enzyme 2 (ACE2) as the primary receptor [13,14]. ACE2 is widely expressed across organs, including the respiratory tract and brain, which influences infection patterns. The viral S protein facilitates attachment and fusion with host cells, requiring ACE2 binding and priming by factors such as Transmembrane Serine Protease 2 (TMPRSS2) and neuropilin-1 (NRP-1). The S protein undergoes cleavage into S1 and S2 subunits, enabling membrane fusion and viral genome entry. ACE2-independent infection appears limited in vivo, and ACE2 receptor-mediated infection is the dominant pathway for SARS-CoV-2 infection [15] (Figure 1). Certain variants (e.g., those with the E484D mutation) can use alternative receptors like Transmembrane Protein 106B (TMEM106B) and heparan sulfate, particularly in brain organoids [15]. In liver infection studies, researchers found that hepatic parenchymal cells, despite low ACE2 expression, can be infected by SARS-CoV-2 primarily via the asialoglycoprotein receptor 1 (ASGR1). In Huh-7 hepatocyte-derived carcinoma cells, simultaneous knockout of ACE2 and ASGR1 completely blocked pseudovirus infection. Conversely, immortalized THLE-2 cells and primary hepatocytes, both with minimal ACE2 expression, remained susceptible to infection. Targeting ASGR1 with siRNA or antibodies significantly reduced infection rates, confirming ASGR1 as the dominant entry pathway in these cells. Further ACE2 knockdown had a negligible effect due to its near-undetectable baseline expression, underscoring ASGR1’s critical role in hepatocyte infection [16].

Given the potential intersection of SARS-CoV-2 and the Human Immunodeficiency Virus-1 (HIV-1) in the CNS, it is crucial to explore in greater depth the possible impacts of these two viral infections and the resulting neurological dysfunction in People with HIV (PWH). In this review, we examine the current research on SARS-CoV-2 infections and COVID-19 morbidity and mortality in PWH to identify key features of synergy and gaps in knowledge. We describe many of the findings on SARS-CoV-2 and the brain, as the effects of HIV-1 have been long studied and are summarized, in comparison to SARS-CoV-2, in Table 1. A comprehensive overview of studies exploring the interplay between SARS-CoV-2 and HIV-1 infections within the human brain is described in the following broad areas (Table 2): (1) COVID-19 Incidence and Outcomes in PWH, (2) COVID-19 Vaccine Responses in PWH, (3) SARS-CoV-2 Infection of the CNS Cells, (4) COVID-19 Neuropathology, (5) Neuropsychological Impacts in PWH and (6) Long-COVID in PWH.

## 2. COVID-19 Incidence and Outcomes in PWHs

Unlike SARS-CoV-2, HIV-1 is initially found in the brain in the perivascular macrophages, and subsequently, microglia become infected. Both macrophages and microglia can produce infectious HIV-1 [71,72]. HIV-1 and SARS-CoV-2 are unique viruses with different modes of transmission and mechanisms of infection. HIV-1 is primarily transmitted through sexual contact, blood-to-blood contact, or perinatal transmission, while SARS-CoV-2 mainly spreads through respiratory droplets.

NeuroHIV broadly refers to the effects of HIV-1 infection on the CNS, including the brain, spinal cord, and sometimes the peripheral nervous system. A common manifestation of neuroHIV is known as HIV-Associated Neurocognitive Disorder (HAND), which encompasses a range of cognitive problems from mild difficulties in memory, attention, and executive functioning to more severe neurocognitive impairment [73,74]. Despite Antiretroviral Therapy (ART) and effective viral suppression, HIV-1 can remain in the brain, maintaining a life-long latent viral reservoir. This leads to chronic, low-level neuroinflammation, immune activation, and neuronal damage. Therefore, even virally-suppressed PWH on ART are at risk of long-term neurological effects, which may slowly accumulate over the lifespan, and potentially worsen with age and comorbidities [75,76]. Unlike SARS-CoV-2, there is no vaccination for HIV-1 infection. However, the continued success of ART has led to increased life spans and better management of the disease, including neurological complications. The key features of both neuroHIV and COVID-19 are highlighted in Table 1.

Since HIV-1 transmission primarily occurs through sexual contact, while SARS-CoV-2 spreads via respiratory droplets requiring close physical proximity, any compromise in prevention efforts could adversely affect the incidence and outcomes of both infections. During the COVID-19 pandemic, HIV-1 prevention efforts were disrupted. HIV-1 PreExposure Prophylaxis (PrEP) usage and testing were reduced in both New York City and Atlanta in the United States of America (USA). In New York City, among 13–24-year-olds, PrEP usage declined to 60% of pre-COVID levels. At the same time, there was a major wave of COVID-19 in both cities [30]. In the USA, the pandemic led to a 22.0% reduction in PrEP prescriptions and a 25.0% decrease in new PrEP users, particularly affecting younger individuals and those with commercial insurance [31]. Changes in PrEP access were associated with a higher risk of HIV-1 seroconversion among Black and Hispanic/Latino sexual minority men and gender-diverse individuals. A study found that individuals reporting changes in PrEP access during the pandemic had an adjusted odds ratio of 2.80 for HIV-1 seroconversion, indicating a substantial increase in risk compared to those with stable access. This study highlighted that increased sexual activity and the number of sexual partners during this period were also associated with higher sexually transmitted infection rates, further complicating the risk landscape for HIV-1 [32]. A similar trend of decline in PrEP usage was reported from Melbourne, Australia [33]. Echoing trends from the rest of the world, during lockdowns in China, the proportion of PrEP users decreased from 97.9% to 64.3%, and the proportion of poor PrEP adherence increased from 23.6% to 50.1%. After the lockdown restrictions were lifted, high-risk sexual behavior increased, and the proportion of individuals tested for HIV-1 decreased from 50.1 to 25.9% [34]. Similar trends were noted in France, with only 62% of participants continuing to use PrEP during the lockdown, and there was a subsequent increase in sexual risk-taking behaviors [35]. Overall, the COVID-19 pandemic disrupted the rising trend in PrEP usage, emphasizing the necessity for novel strategies to maintain access to HIV-1 prevention services during emergencies [31,36].

While PrEP usage and adherence decreased, and sexual risk-taking behavior increased, COVID-19 incidence in PrEP users was comparable to that of the general population [35,36]. In a German Men who have Sex with Men cohort of PrEP users, anti-N SARS-CoV-2 IgG was detected at a seroconversion rate of 7.3%. The seroconversion rate was comparable to the proportion of positive cases, which stood at 6.5% in Munich by 2021 [77]. In the French cohort study, SARS-CoV-2 incidence rates were similar among PrEP users (14.8%), the general population (19.1%), and PWH (15.6%) [78]. These studies led to the perception that PrEP users and PWH were not experiencing a higher incidence of COVID-19 than the general population, indicating their risk aligned more closely with everyone [34].

PWH who are virologically suppressed on ART do not appear to have substantially higher susceptibility to SARS-CoV-2 infection than People without HIV (PWoH). However, once infected, PWH exhibit a consistently elevated risk of adverse outcomes [79]. Global guidance and recent meta-analytic syntheses indicate higher odds of severe disease and death, with the World Health Organization reporting a ~38% increased risk of severe or fatal COVID-19 among PWH and newer evidence showing elevated adjusted mortality [80]. In population-level cohorts, risk estimates are concordant: New York City surveillance from 2020 found ~30% higher hospitalization and mortality among PWH, and a nationwide Spanish study (2020–2022) identified HIV-1 infection as an independent risk factor for in-hospital death [41,81]. Early meta-analysis from 2021 further suggests increased infection risk and higher mortality among PWH versus HIV-negative comparators [82].

The drivers of this elevated severity are multifactorial. Traditional risk factors for poor COVID-19 outcomes (older age, male sex, cardiometabolic comorbidities) are more prevalent among PWH and are independently associated with death in pooled analyses [83]. HIV-1-specific factors such as low CD4 counts and unsuppressed viral load are strongly associated with hospitalization and death in PWH with COVID-19 [41,79]. Social determinants of health (e.g., structural racism, poverty, housing instability, limited access to care) likely compound these risks by increasing exposure and attenuating timely access to prevention and treatment services [41]. Collectively, these findings support prioritization of PWH for preventive and early therapeutic interventions, particularly those with immunologic vulnerability or significant comorbidity burdens.

The heightened vulnerability of PWH to respiratory infections such as influenza and pneumococcal pneumonia, COVID-19 incidence, and subsequent outcomes led to many studies. In terms of COVID-19 outcomes in PWH, in the early stages of the pandemic, a clear link was not established between HIV-1 infection and increased COVID-19 severity or mortality. Numerous studies attempted to resolve whether HIV-1 infection itself, co-morbid conditions, and/or ART influence susceptibility to SARS-CoV-2 infection and COVID-19 disease outcomes. The answers have not always been unequivocally clear due to the underlying complexity of the disease and variations in the study populations. Chronic HIV infection may exacerbate COVID-19 severity through synergistic immune dysregulation. Although epidemiologic data are heterogeneous, advanced HIV-1 disease characterized by CD4+ T-cell depletion, persistent inflammation, and impaired type I interferon signaling correlates with increased hospitalization and mortality. HIV-1-associated comorbidities, including cardiometabolic disorders, further amplify risk. ART partially restores immune competence and improves clinical outcomes, though its direct antiviral activity against SARS-CoV-2 remains inconclusive. Current evidence supports the safety of mRNA and adenoviral vector vaccines in PWH, with robust seroconversion observed despite attenuated antibody titers in subsets with immunosuppression. These findings underscore the imperative to prioritize PWH for vaccination, maintain uninterrupted ART, and pursue longitudinal studies to define immune durability and optimize prophylactic strategies in this population [84].

Severity of COVID-19 outcomes was associated with comorbidities and social determinants of health. Additional studies now point to low CD4 count or unsuppressed HIV-1 RNA as an independent risk factor for COVID-19 severity [37,38,39,40]. In a New York cohort study at the onset of the pandemic, the risk of hospitalization and death among PWH with COVID-19 was nearly 30% higher compared with PWoH [41]. Results from a nationwide Swedish study indicated that HIV-1 infection was not a risk factor in hospitalized patients for developing severe COVID-19. Almost all PWH in this study were well-controlled with undetectable HIV-1 RNA and high CD4 T-cell counts (median = 560 cells/μL) [38]. In contrast, the nationwide study of COVID-19 outcomes in PWH from the Netherlands included both well-controlled and uncontrolled PWH. In this study, they concluded that the risk of severe COVID-19 outcomes increased in individuals with uncontrolled HIV-1 replication, low CD4+ T cell count, and prior AIDS diagnosis, independently of general risk factors such as higher age and comorbidity burden [39]. These observations were reinforced by an additional study from the USA, which concluded that PWH with low CD4 T cell counts had worse COVID-19 outcomes compared with HIV-1-negative individuals. Well-controlled PWH in this study had similar or better outcomes compared to PWoH [40].

Emerging evidence suggests that asymptomatic SARS-CoV-2 infection is common among PWH, though not necessarily higher than in HIV-1-negative populations. In the REPRIEVE trial, which included 2464 PWH, 304 participants had confirmed SARS-CoV-2 infection, and 60% of these cases were asymptomatic [42]. This proportion is consistent with general population estimates, where asymptomatic infection rates range from 40% to 50% [43,85,86]. These findings underscore that while asymptomatic infection is frequent among PWH, it does not appear to represent a unique epidemiological pattern.

Resource-limited countries, for example, in sub-Saharan Africa with high prevalence of HIV-1 infections, COVID-19 had a direct impact on PWH living in those regions, and a secondary response resulting from the shutdowns in place during the pandemic. In a global meta-analysis, researchers inferred that HIV-1 infection may increase the severity of COVID-19 in Africa [87]. However, for much of sub-Saharan Africa, the overall burden of COVID-19 was relatively small compared with long-standing endemic diseases, including TB, malaria, and HIV/AIDS [88]. Thus, while COVID-19 added to the risk, perhaps for PWH, it did not become the dominant disease burden compared to existing chronic infectious diseases. The lockdown-driven disruption of healthcare services for PWH and the diversion of resources to COVID-19 were, to some extent, mitigated by multi-month dispensing of ART, home-based or self-testing for HIV, or remote counseling. These measures were expected to reduce the need for frequent clinic visits and minimize COVID-19 risk [89].

Researchers have looked at whether being on ART for HIV-1 changes outcomes if someone then gets COVID-19. A cohort study of 77,590 HIV-positive persons in Spain found that those on the nucleos(t)ide reverse transcriptase inhibitors (NRTIs) tenofovir disoproxil fumarate (TDF)/emtricitabine (FTC) ART backbone (TDF/FTC) had lower rates of COVID-19 diagnosis and hospitalization than people on some other ART regimens. However, additional investigation in HIV-1 PrEP studies and randomized trials in PWoH is recommended [90]. In another cohort study from France, the risk of symptomatic COVID-19 appeared similar in PWH, PrEP users, and the general population [78].

COVID-19 treatments such as Paxlovid (nirmatrelvir/ritonavir) or dexamethasone can interact with ART in PWH. However, ART doesn’t need to be changed, and it depends a lot on which antiretrovirals the person is taking. PWH on stable ART, using Paxlovid for COVID-19, do not require preemptive ART dose adjustments [91]. Ritonavir inhibits liver enzyme CYP3A4, which in turn can boost blood levels of protease inhibitors and some integrase inhibitors (e.g., Bictegravir, dolutegravir, and raltegravir), with minimal impact on pharmacokinetics, allowing standard dosing without adjustment, while encouraging clinical monitoring for tolerability and virologic control [92]. Combining Paxlovid and cabotegravir may significantly reduce cabotegravir blood levels, potentially compromising the effectiveness of the ART. Cabotegravir is primarily metabolized by UGT1A1, which is induced by Ritonavir, and it can therefore accelerate cabotegravir metabolism, lowering its plasma concentration. Concomitant use of Paxlovid and cabotegravir is not recommended, as the HIV treatment effectiveness is diminished. Furthermore, dexamethasone is metabolized through CYP3A4, so co-administration with ritonavir can increase dexamethasone levels, raising a theoretical risk of steroid overexposure, although with the short-course, low-dose dexamethasone used for COVID, the real-world risk is considered low. Overall, for PWH on the standard ART regimen (e.g., with an integrase inhibitor or NRTIs, not cabotegravir), they can safely receive Paxlovid and continue ART without changes. If their ART includes cabotegravir or other drugs that might be particularly affected by CYP3A/P-gp changes, there is a risk that Paxlovid might lower ART drug levels or otherwise disrupt HIV control. A careful evaluation by the HIV specialist would be required [91].

In summary, factors such as older age, low CD4 cell counts, detectable viral loads, and comorbidities such as hypertension, diabetes, and chronic lung disease may further increase the risk. Effective ART that suppresses the HIV-1 viral load and preserves immune function may mitigate the risk of severe COVID-19 outcomes. Factors such as the prevalence of HIV-1, the availability of healthcare resources, and public health interventions may influence COVID-19 outcomes.

## 3. COVID-19 Vaccine Responses in PWH

Although highly effective vaccines have been developed for SARS-CoV-2, similar success has not been achieved for HIV-1. This disparity reflects fundamental differences in viral biology: SARS-CoV-2 presents relatively stable antigenic targets, enabling rapid vaccine development, whereas HIV-1 exhibits extreme genetic variability, integrates into host DNA, and employs sophisticated immune-evasion strategies that have thwarted vaccine efforts for decades [93,94]. The availability of SARS-CoV-2 vaccines has significantly reduced viral persistence and systemic inflammation. These factors are implicated in neurological complications and Long-COVID. For example, studies indicate that in adults, receiving vaccination before infection was linked to a 69% reduction in Long-COVID symptoms at 90 days post-infection [95,96]. Additionally, mRNA vaccination has been shown to decrease the accumulation of spike protein in brain border tissues, potentially mitigating chronic brain inflammation [97]. In contrast, the absence of an HIV-1 vaccine contributes to chronic infection and persistent immune activation. Despite suppressive ART, PWH remain at high risk for neurocognitive disorders driven by innate immune memory and neuroinflammation [98,99,100]. These observations underscore the critical role of early immunological control, ideally via vaccination, to mitigate long-term brain effects of viral infections.

PWH exhibit suboptimal response to COVID-19 vaccines, shown to correlate with HIV-1 infection, triggering immune-senescence and perturbations in B-cell activation [44]. The vaccines were well-tolerated in PWH, but the immunological outcomes were poorer, especially with non-mRNA vaccines, and low CD4+ T cell counts. PWH were 3% less likely to seroconvert and 5% less likely to demonstrate neutralization responses after a primary vaccine schedule [45]. In a study evaluating the immunogenicity of a bivalent BA.1 COVID-19 booster vaccine in well-controlled ART-treated PWH with undetectable HIV-1 viral load and high CD4+ T cell levels, the immunogenic responses were comparable to those of PWoH. However, T-cell responses waned faster after 90 days in PWH compared to PWoH [46]. Researchers have shown that PWH with CD4+ T-cell counts <200 cells/mm^3^ may have reduced immune responses to mRNA vaccines, with one-third showing low antibody levels and diminished neutralization activity [47]. However, individuals with PWH receiving inactivated COVID-19 vaccines demonstrate enhanced CD4+ T-cell subsets and neutralizing antibody responses, with increased Treg subpopulations and satisfactory antibody levels post-vaccination [48]. Another study focused on older PWH showed that they retained SARS-CoV-2 anti-Spike IgG and T-cell responses 6 months post-vaccination, suggesting some degree of long-term protective immunity [49].

PWH were underrepresented in COVID-19 vaccine efficacy trials. In some trials, PWH on stable ART with well-controlled viremia were included. However, individuals with advanced HIV-1 infection were excluded. PWH represented only 1% of participants for Phase 2/3 trials testing the mRNA-1273/Moderna, BNT 162b2/Pfizer-BioNTech, ChAdOx1/AstraZeneca, NVX-2373/Novavax, and AD26.COV2.S/Janssen vaccine efficacy, with other major vaccine trials excluding or not reporting numbers of PWH. The Novavax trial in South Africa results for vaccine efficacy were demonstrably lower in those seronegative for SARS-CoV-2 when PWH were included in the analyses (60.1% for PWoH vs. 49.4% when PWH were included). However, low numbers of PWH generally preclude robust subgroup analysis to detect differences in vaccine efficacy, rendering the question of vaccine efficacy in this group unresolved. To date, the largest study of PWH was a phase 3 open-label implementation trial of a single dose of Ad26.COV2.S/Janssen vaccine in 477,102 health care workers in South Africa, of whom 8.3% were PWH, mostly women. Similar effectiveness was demonstrated for health care workers with HIV-1 compared to HIV-1-uninfected health care workers for hospital admissions, including critical care. A higher number of COVID-19 deaths in vaccinated PWH compared to the PWoH group was reported, though total deaths were low because the vaccine maintained good efficacy against fatal COVID-19; therefore, these analyses are limited.

## 4. SARS-CoV-2 Infection of the CNS

SARS-CoV-2 exhibits neurotropism, infecting diverse CNS cell types and contributing to neuropathology through ACE2, TMPRSS2, and NRP-1 receptors. ACE2 expression was assessed in 85 human tissue samples across 21 brain regions. Elevated levels of ACE2 were detected in the amygdala, cerebral cortex, and brainstem, while the pons and medulla oblongata had the highest expression of the receptor [101]. In another study, investigators examined human brain tissues from 18 subjects for expression of ACE2 and TMPRSS2 within the brain-stem. They detected expression of these receptors in neuronal and glial cells within the brain stem [102]. Furthermore, Haverty and coworkers (2024) analyzed the frontal cortex and medulla oblongata from five healthy human donors for expression of the ACE2, together with TMPRSS2. Low-level cytoplasmic expression of both receptors was observed in cells morphologically consistent with neurons and astrocytes in both brain areas examined from all five donors. In a single donor for which choroid plexus was available, choroidal ependymal cells were immunoreactive to both ACE2 and TMPRSS2 [20].

To further elucidate the cellular mechanisms underlying SARS-CoV-2 neurotropism, recent studies have examined receptor expression and viral infectivity across diverse human brain-derived cell types. Malik et al. (2023) utilized human brain-derived astrocytes and pericytes to measure expression of these receptors. In their study, astrocytes expressed ACE2, TMPRSS2, and NRP-1. Pericytes exhibited ACE2 expression comparable to astrocytes, with moderately lower NRP-1 levels [17]. Of note, ACE2 expression in astrocytes is relatively low compared to lung epithelial cells and is limited to a small subset of astrocytes [17]. Haverty et al. (2024) performed in vitro studies with primary human brain-derived neurons, astrocytes, choroid plexus epithelial cells, brain microvascular endothelial cells, brain vascular pericytes, and immortalized human microglia to test viral infectivity. Astrocytes, neurons, and choroid plexus epithelial cells supported SARS-CoV-2 infection, with low levels of infection observed in pericytes. Astrocytes supported the highest level of infection, while microglia and brain microvascular endothelial cells did not support SARS-CoV-2 infection. ACE2 antibody-mediated neutralization in neurons and choroid plexus cells significantly decreased SARS-CoV-2 infection. A similar significant decrease was not observed with astrocytes [20]. Kettunen et al. (2023) utilized a human induced pluripotent stem cell-derived neuron-astrocyte coculture system to assess their infectivity to SARS-CoV-2 and identify entry mechanisms [18]. Immunofluorescence and RNA quantification confirmed productive infection in a small subset of neurons, evidenced by cytoplasmic double-stranded RNA and nucleocapsid protein, with viral RNA levels increasing in culture supernatants by 48 h post-infection. Astrocytes remained resistant under identical conditions. Neutralizing antibodies against ACE2 significantly reduced infection, indicating ACE2 dependence, while TMPRSS2 inhibitors had no effect, demonstrating TMPRSS2 independence. Pharmacologic blockade of endosomal maturation completely abrogated infection, supporting an endosomal route of viral entry. These findings highlight that SARS-CoV-2 infects human neurons via ACE2-mediated, TMPRSS2-independent endosomal uptake and suggest endosomal trafficking as a potential therapeutic target. Astrocytes were not infected in this study primarily because they lacked the necessary conditions for productive SARS-CoV-2 replication. Human-induced pluripotent stem cell-derived brain organoids have been employed to investigate SARS-CoV-2 neurotropism. Viral spike and nucleocapsid proteins were detected within infected organoids, with pronounced colocalization of spike protein in GFAP-positive astrocytes, identifying astrocytes as a principal target. Neurons and neural progenitor cells exhibited lower levels of infection. NRP1 knockdown via siRNA or antibody-mediated neutralization significantly reduced astrocyte infection in the organoids. Additionally, productive viral replication in astrocytes peaked at 72 h post-infection, creating a pro-inflammatory milieu that likely contributed to the dysfunction and death of uninfected bystander neurons [18]. Collectively, these findings underscore the complexity of SARS-CoV-2 neurotropism, revealing cell-type-specific susceptibility and entry mechanisms that highlight ACE2-dependent, TMPRSS2-independent pathways as critical targets for therapeutic intervention [21].

SARS-CoV-2 exhibits marked neurotropism, with astrocytes emerging as a principal target within the human brain. Postmortem examinations of COVID-19 brains confirmed the presence of SARS-CoV-2 RNA and spike protein in astrocytes, with double-stranded RNA indicating active replication [19]. Approximately two-thirds of spike-positive cells were GFAP+ astrocytes, while neurons exhibited lower infection rates and microglia remained refractory to direct infection. Complementary ex vivo studies using human cortical slices demonstrated similar tropism, with astrocytes accounting for the majority of infected cells. Mechanistically, viral entry into astrocytes occurs predominantly via NRP1, bypassing the canonical ACE2 pathway, which is minimally expressed in these cells. This ACE2-independent route underscores the adaptability of SARS-CoV-2 in exploiting alternative receptors for CNS invasion [30]. The functional consequences of astrocyte infection could result in their metabolic rewiring, reducing levels of neurotransmitter precursors and key metabolites essential for neuronal support, and contributing to neuronal dysfunction. Clinically, these cellular changes align with imaging and cognitive findings, implicating astrocyte-driven neuroinflammation as a central mechanism underlying both acute neurological symptoms and long-term sequelae, including cognitive decline and mood disturbances [30,75]. Collectively, these findings highlight the need for targeted investigations into astrocyte-mediated pathways of SARS-CoV-2 neuropathogenesis. Understanding receptor-specific entry mechanisms and downstream inflammatory cascades may inform therapeutic strategies aimed at mitigating CNS injury and preserving cognitive function in COVID-19 survivors.

The detection of SARS-CoV-2 in the CNS of patients has not been consistent. In one study, researchers utilized 44 autopsies of people who had died with COVID-19 conducted between 26 April 2020 and 2 March 2021. This cohort consisted entirely of unvaccinated individuals. Out of the 44 cases, 42 were confirmed to be SARS-CoV-2 PCR positive before death, while 2 cases were confirmed postmortem. The researchers employed various techniques to detect SARS-CoV-2 in brain tissues, including droplet digital polymerase chain reaction for quantifying the nucleocapsid (N) gene, in situ hybridization for RNA detection, and immunofluorescence and chromogenic immunohistochemistry for protein detection. Researchers confirmed the presence of SARS-CoV-2 RNA and protein in specific brain regions. Autopsy cases in this study were categorized by duration of infection. The early, mid, or late cases had a duration of infection of ≤d14, d15-d30, or ≥d31, respectively. Notably, the hypothalamus and cerebellum showed positive results in one of the three early cases, while the cervical spinal cord and basal ganglia were positive in two of the six later cases. The staining patterns observed were consistent with neuronal infection. The researchers demonstrated that SARS-CoV-2 was not only present but also capable of replication within the brain. They recovered replication-competent virus from the thalamus of the early case at day 13 post-symptom onset, indicating active viral replication in the CNS. Alongside the hypothalamus, the cerebellum also showed positive detection of SARS-CoV-2 in the same early case. In one of the late cases, the cervical spinal cord was found to be positive for SARS-CoV-2. Another late case revealed positive detection of the virus in the basal ganglia, which are involved in movement regulation and various cognitive functions [29]. These findings underscore the importance of understanding the implications of SARS-CoV-2 infection in the brain, particularly regarding potential long-term effects and the mechanisms underlying neurological symptoms observed in COVID-19 patients.

Human postmortem studies consistently demonstrate that microglia are not directly infected by SARS-CoV-2, but exhibit profound activation in response to systemic and CNS inflammation. Autopsy analyses reveal diffuse microglial activation and clustering, particularly in white matter, brainstem, and cerebellar regions, accompanied by perivascular macrophage infiltration. These changes include downregulation of purinergic receptor P2Y12R, a key regulator of microglial homeostasis, correlating with increased neuroinflammatory markers such as IL-1, IL-6, and MCP-1 in cerebrospinal fluid and brain tissue. Prominent astrogliosis and microglial nodules in hindbrain regions further underscore their role in neurovascular inflammation and neuronal injury. Collectively, these findings indicate that while microglia remain refractory to direct viral infection, they contribute significantly to neuropathology through immune dysregulation and inflammatory cascades in COVID-19 brains [18,20,22]. Similar changes in microglia are found in the brains of people with HIV-1 (PWH) [103].

SARS-CoV-2 can enter the CNS through multiple routes, including the olfactory bulb, the blood–brain barrier, and the blood-CSF barrier [23,24]. Once inside the CNS, the virus can spread to and infect various brain regions [20,25,104]. One potential entry site is the choroid plexus, which produces the CSF. Viral RNA and proteins have been detected in the choroid plexus epithelial cells of COVID-19 patients. This suggests that the virus directly infects these cells and may lead to the disruption of the blood-CSF barrier and the spread of the virus within the CNS [23,24]. Studies on SARS-CoV-2 infection of the olfactory bulb have produced mixed results. In non-human primates, the virus induced inflammation and was detected in olfactory bulbs, supporting a potential intracranial route via the olfactory epithelium (OE). Viral RNA and nucleocapsid protein were found in olfactory neurons and axons, indicating spread from the OE to the brain [26]. Conversely, human autopsy studies detected SARS-CoV-2 RNA in lungs but not in olfactory bulbs, despite ACE2 receptor RNA being present in both tissues, with higher expression in lungs [27]. These findings suggest that COVID-19-related anosmia may not result solely from direct olfactory bulb infection. In golden Syrian hamsters, SARS-CoV-2 infected multiple OE cell types, including sustentacular cells and olfactory sensory neurons, causing severe epithelial damage and apoptosis, while olfactory bulbs remained free of viral antigens [28]. Collectively, evidence points to OE infection and local tissue destruction as primary drivers of olfactory dysfunction rather than direct bulb involvement.

## 5. COVID-19 Neuropathology

The CNS infection of HIV-1, the resultant pathology, and disease manifestation are well-documented [73,105]. HIV-1 invasion occurs during the early stages of the infection, and CNS disorders in PWH can be known by the terms HAND or, as in a recent proposal, HIV-1-associated brain injury (HABI) [106,107]. However, the CNS interface of HIV-1 and SARS-CoV-2 remains undercharacterized. COVID-19-related neuropathology (microglial activation, endothelial dysfunction, cerebrovascular injury) is well documented in the general population and plausibly relevant to PWH given chronic HIV-1-related immune activation [108]. Yet direct demonstration of synergistic CNS effects in co-infected individuals is limited [109]. Evidence leveraging large electronic health record datasets suggests excess risk of acute and lingering neurological complications in PWH compared with HIV-1-negative individuals following COVID-19, but confirmatory neuroimaging and CSF biomarker studies are scarce [109]. Mechanistically, SARS-CoV-2’s neurotropic potential and capacity for neuroinflammation raise concerns about exacerbation of HAND, warranting targeted evaluations of microglial priming, endothelial integrity, and blood–brain barrier disruption in PWH post-COVID-19 [108].

One of the earliest COVID-19 autopsy case reports was published for a PWH from South Africa. HIV-1 diagnosis was recent, and as a result, uncontrolled with a low CD4+ T cell count and high HIV-1 viral load. At autopsy, the brain of this individual was unremarkable [50]. Although COVID-19 autopsy reports are abundant, PWH were not identified, nor unique brain pathology attributed to them. This presents a huge knowledge gap and is open to speculation.

A study on 17 patients from Germany who succumbed to COVID-19 compared 5 control patients to characterize the COVID-19 brain pathology. Comorbidities listed for the subjects in this study were the typical, well-known risk factors for COVID-19, such as Diabetes mellitus type 2, coronary heart disease, and nicotine abuse. However, HIV-1 infection status was not mentioned as a co-morbid condition for any of the study patients. Immunohistochemical analysis with CD68 was used to detect diffuse parenchymal microglial activity, pronounced perivascular macrophage activation, and macrophage clusters. Microglial and macrophage activation was observed in the white matter in the brain stem and cerebellar areas. A diffuse microglial pattern was observed in the cerebellar nuclei, the white matter areas of the cerebrum, and the brain stem areas surrounding the pontine nuclei. The perivascular macrophage component showed the same distribution across the analyzed regions of the brain but had less variation in intensity across patients. Lesion patterns did not correlate with disease severity. Macrophage clusters were indicative of advanced microglial and macrophage activation. Commercially available antibodies for the detection of SARS-CoV-2 did not indicate the presence of viral proteins in the examined brain tissues of COVID-19 subjects [51].

Additional information for patterns of gross and microscopic neuropathological autopsy findings in the brains of COVID-19 patients comes from 32 autopsies of COVID-19 suspected patients from California, USA. In this study, they accounted for the presence of cerebral edema (CE), cerebral cortical atrophy (CCA), chronic cerebrovascular disease (CCD), cerebral ischemic injury (CII), cerebral inflammation (CIN), and/or cerebral parenchymal hemorrhage (CPH). CE was the most common finding present in more than half of their cases (71.9%), followed by CII (40.6%), CCA (28.1%), CPH (9.4%), CIN (9.4%), and CCD (6.3%). CE, CCA, and CII diagnoses were significantly associated with age. The study included 3 fetal cases, and they still exhibited the same gross microscopic neuropathological findings that were observed in adults. The cases included in this study had a positive polymerase chain reaction SARS-CoV-2 test at autopsy on post-mortem nasopharyngeal swab samples. No information about the HIV-1 infection status of any of the patients was mentioned [52]. However, given the findings related to COVID-19 only, if there were PWH in this cohort, their neuropathological findings were unlikely to differ from those of the rest of the cohort.

The involvement of microglial dysfunction was highlighted in the examined autopsy tissues of COVID-19 patients. For this study, 11 patients who had died from COVID-19 were included, along with 9 patients in the control group. Most of the COVID-19 patients included in the study were unvaccinated. Inclusion of non-COVID-19 cases allowed exploration of links between systemic inflammation, microglial dysfunction, and neurological outcomes associated with the disease. Like other studies focusing on the COVID-19 neurological outcomes, information regarding the HIV-1 infection status of the patients was not included in the research. Nonetheless, this study highlights the relationship between COVID-19, microglial dysfunction, and systemic inflammation. There were significant changes in the microglial cells in COVID-19 patients, particularly in central autonomic nuclei, indicating a profound impact on brain immune response. A notable finding was the downregulation of the purinergic receptor P2Y12R in microglia across various brain regions affected by COVID-19. P2Y12R receptor is crucial for microglial responses to injury and infection, and its loss correlates with increased viral load of SARS-CoV-2, suggesting a direct link between viral presence and microglial state. In this study the SARS-CoV-2 nucleocapsid was detected at high levels in the olfactory bulb and cranial nerves. In the parts of the brain that were examined, it was mostly localized to blood vessels and not found inside neurons. COVID-19 neuropathology also included loss of vascular integrity, neuronal injury, and changes in synaptic structures. A strong correlation was observed between inflammatory cytokines, such as interleukin-1, interleukin-6 (IL-6), and monocyte chemoattractant protein 1 (MCP-1), in the CSF and brain tissues, indicating neurovascular inflammation [53].

SARS-CoV-2 infection may disrupt the blood–brain barrier, allowing immune cells and inflammatory mediators to enter the brain. Gene expression changes associated with blood–brain barrier disruption and endothelial cell activation have been observed in COVID-19 patients, indicating potential mechanisms of neuroinvasion and neuroinflammation. A study on neurovascular injury in COVID-19 examined the neuropathological changes, focusing on neurovascular injury and the role of complement activation and inflammation. The researchers included adult patients who died during the first wave of the pandemic, specifically from March to July 2020. All patients had confirmed SARS-CoV-2 infection through ante-mortem or post-mortem testing. All patients displayed multifocal vascular damage characterized by serum protein leakage into the brain parenchyma. This was associated with widespread activation of endothelial cells, indicating significant vascular pathology. The presence of platelet aggregates and microthrombi was noted along the vascular lumina, suggesting a potential mechanism for neurovascular injury. Immune complexes activating the classical complement pathway were found on endothelial cells and platelets. This suggests that the immune response may contribute to the observed vascular and neuronal damage. The perivascular regions were predominantly infiltrated by macrophages and CD8+ T cells, with only rare CD4+ T cells and CD20+ B cells present. With regard to neuroinflammation, prominent astrogliosis was observed in perivascular regions, along with microglial nodules, particularly in the hindbrain. This neuroinflammatory response was associated with focal neuronal loss and neuronophagia. The findings suggest that antibody-mediated cytotoxicity against endothelial cells may initiate a cascade leading to vascular leakage, neuroinflammation, and neuronal injury [54].

Patients with COVID-19 experience a higher frequency and severity of abnormal blood clots compared to those with other common respiratory viruses. Fibrinogen, a structural component of blood clots, interacts with the S protein and is abundantly deposited in the brains of COVID-19 patients. This biomarker is associated with cognitive deficits in individuals who have contracted COVID-19. The conversion of fibrinogen to fibrin exposes a cryptic inflammatory epitope that is linked to a potent inflammatory response. This response can exacerbate the severity of COVID-19, suggesting that the fibrinogen-fibrin pathway plays a crucial role in the disease’s pathology. To explore the role of fibrinogen in COVID-19 neuropathology, the researchers utilized data from pathology, radiology, and serology in patients with both acute COVID-19 and long COVID to establish a connection between fibrin and neuropathology. They noted that fibrin deposition in the brain correlates with neurovascular injury and reactive microglia, which are indicative of neuroinflammation and neuronal loss. Additionally, the study employed genetic loss-of-function, pharmacological, and transcriptomic studies in three animal models of COVID-19. These models helped to elucidate the role of fibrinogen in driving inflammation and neuropathology during SARS-CoV-2 infection. The findings indicated that fibrinogen is a key driver of these pathological processes. The research highlights that the mechanisms involving fibrin and its effects on the immune response may contribute to the persistent neurological symptoms observed in long COVID patients. The correlation between increased plasma fibrinogen levels and cognitive deficits further emphasizes the importance of fibrin in the long-term consequences of COVID-19 [55].

In contrast to the previously mentioned autopsy-based studies aimed at understanding the neuropathology of COVID-19, the investigators from the University Medical Center Freiburg, Germany, analyzed a cohort of living patients diagnosed with neuro-long-COVID-19, following the World Health Organization’s Post-COVID-19 Condition criteria. This cohort was essential for understanding the neurological impacts of COVID-19, as traditional autopsy studies were limited due to the lack of available autopsy cases for neuro-Long-COVID-19 patients. For comparison, healthy controls were included alongside post-COVID-19 patients. The researchers noted a significant increase in specific microglial markers, indicating a shift towards innate immune activation, which could be linked to neurological changes. The results indicated that individuals with post-COVID-19 showed a high percentage of certain immune cell markers, including TMEM119, P2RY12, CD68, Iba1, HLA-DR, CD11c, and SCAMP2, which were clustered in prototypical cellular nodules. In contrast to acute SARS-CoV-2 infection, the frequency of CD8+ parenchymal T cells was found to be lower in post-COVID-19 patients. This shift indicates a potential transition from adaptive to innate immune responses, which may contribute to the neurological changes observed in these individuals. The study concluded that there is notable dysregulation of the innate immune system in the brains of COVID-19 survivors who did not exhibit neurological symptoms during their lifetime. This finding suggests that even in the absence of overt neurological issues, immune alterations may still exist and could have implications for long-term health. The study also analyzed CSF samples from living patients diagnosed with neuro-Long-COVID-19. However, the researchers did not detect any distinct disease-specific patterns in these analyses, indicating that further investigation is needed to identify potential biomarkers associated with long-term neurological effects. In the autopsy cohort, typical neuropathological hallmarks of neuronal degeneration were not observable, suggesting that while immune dysregulation is present, it may not correlate with visible neuronal damage in these patients. These results highlight the complex relationship between immune responses and neurological health in individuals recovering from COVID-19, emphasizing the need for further research to understand the long-term implications of these findings [56]. The provided contexts do not contain any specific information regarding the HIV-1 status of the study cohort. Therefore, it is unclear whether the participants in the study were tested for HIV-1 or if their HIV-1 status was a factor in the research.

A study investigated the mid-term effects of COVID-19 on the brain, particularly focusing on individuals who experienced anosmia during the acute phase but were never hospitalized. The research included 43 individuals, divided into two groups: those who had been infected with SARS-CoV-2 (COV+, *n* = 22) and those who had not (COV−, *n* = 21). The groups were matched for age and sex. Participants underwent 3T magnetic resonance imaging to assess various brain metrics, including gray matter volume, white matter hyperintensity volume, and microstructural integrity (measured by fractional anisotropy, mean diffusivity, axial diffusivity, and radial diffusivity). The study found no significant differences between the COV+ and COV− groups regarding gray matter volume, white matter hyperintensity volume, and cerebral blood flow. With regard to microstructural changes in the brain, the COV+ group exhibited local white matter microstructural alterations, including lower fractional anisotropy, indicating reduced integrity of the white matter tracts and higher radial diffusivity, indicative of damage or changes in the microstructure. The results suggest that even individuals who experience mild-to-moderate COVID-19 without hospitalization may still suffer from microstructural brain changes [57].

Methodologically, many discussions of COVID-19 neuropathology in the HIV-1 context extrapolate from studies where HIV-1 status was unrecorded, potentially over-generalizing mechanisms from HIV-1-negative cohorts. Recent reviews explicitly note the scarcity of neuroimaging, blood, and CSF biomarker studies capable of establishing true pathophysiological “synergy” in the CNS [109]. More robust prospective designs with matched HIV-1-negative comparators and multimodal biomarkers (e.g., CSF cytokines, neurofilament light chain, microglial imaging, endothelial function) are needed to define mechanisms and trajectories [108]. Limitations of the current evidence include reliance on early pandemic datasets (pre-vaccine/early variants) and heterogeneity in confounder adjustment, outcome definitions, and data sources; however, updated meta-analytic syntheses continue to demonstrate elevated adjusted mortality risk among PWH, while cohort studies confirm vaccination benefits for reducing acute severity and post-acute sequelae of SARS-CoV-2 (PASC) [80,110]. In summary, although infection susceptibility in well-controlled PWH is broadly similar to the general population, the risk of severe disease, mortality, and post-acute complications is higher, particularly in those with low CD4 counts, unsuppressed viral load, and multiple comorbidities. CNS synergy remains plausible but insufficiently defined, necessitating rigorous, mechanism-focused studies [108,109].

## 6. Neuropsychological Impairment in PWH

The attribution of neurocognitive symptoms to post-COVID effects in PWH is further complicated by pre-existing neurological conditions common in this population. For example, survivors of cryptococcal meningitis frequently exhibit persistent cognitive impairment, including memory deficits, slowed processing, and executive dysfunction, months after treatment, often linked to elevated intracranial pressure and structural damage [111,112]. Similarly, TB meningitis is associated with prolonged cognitive impairment across multiple domains in nearly half of survivors, with deficits in attention, memory, and processing speed, likely due to meningeal fibrosis and vasculitis [113]. Cerebral toxoplasmosis may cause chronic executive dysfunction and subtle cognitive changes due to persistent gliosis or inflammatory lesions, mimicking sequelae of viral injury [114,115]. Additionally, HAND affects up to 40% of PWH and is characterized by attention deficits, slowed thinking, and motor-skill deterioration. These HAND symptoms overlap strikingly with reported post-COVID cognitive decline [116,117]. These overlapping clinical and imaging findings underscore the necessity of careful differential diagnosis, incorporating neuropsychological testing, neuroimaging, and CSF or biomarker analysis to accurately discern whether observed deficits are attributable to prior conditions or new post-COVID changes.

PWH reported high rates of mental health problems during the pandemic. Multi-level factors were associated with increased psychological distress, including substance use, antiretroviral adherence, social support, financial hardship, and economic vulnerability during the pandemic. PWH used social media as a coping strategy to foster social support to deal with growing mental distress. Increased mental health illnesses were associated with increased substance use. It was also found to be associated with suboptimal medication adherence and ART care engagement. At the individual level, age and gender were significant predictors of mental health outcomes. Several studies found that older (versus younger) PWH were more likely to experience psychological distress during the pandemic [58,59,60]. At the interpersonal level, mental health outcomes were associated with social support and changes in interpersonal relationships in accordance with COVID-19-related measures. Studies found that lower levels of social support were correlated with higher depressive and anxiety symptoms [61,62,63]. The most frequently assessed mental health outcomes were depression (57.8%), anxiety (46.7%), and loneliness (22.2%). Other outcomes included stress, PTSD, and sleep disorders [64]. US adults reported anxiety and depression symptoms during the pandemic, and the rates improved by 2023–2024 [118]. However, the rates of these mental health outcomes were higher in PWH. In summary, the COVID-19 pandemic significantly impacted the mental health of PWH, driven by complex individual, interpersonal, and structural factors, necessitating integrated and multi-faceted interventions to support this vulnerable population and ensure continued HIV-1 care [65]. Well-designed, peer-reviewed studies specifically examining neuropsychological outcomes of COVID-19 in PWH are still emerging. Of note, some studies excluded PWH in their attempt to minimize the influence of confounders [119]. In an interesting study, the Manhattan Brain Bank included PWH but focused on the efficacy of two cognitive testing modalities: “in-person” or “via telephone” during the COVID-19 pandemic. They concluded that telephone-based cognitive screening was a viable alternative to in-person testing for maintaining longitudinal research and monitoring cognitive health in PWH during public health disruptions. While most adapted tasks performed well, some (like verbal memory) showed variability, indicating the need for careful interpretation and further validation [120].

A study aimed to evaluate cognitive functions in PWH both before and after COVID-19 infection, utilizing the Montreal Cognitive Assessment (MOCA) scale, included 116 virally suppressed PWH, with a mean age of 47.6 years, and 91.4% were male [66]. A MOCA score greater than 25 was indicative of normal cognitive performance. A MOCA score ranging from 18 to 25 indicated mild cognitive impairment. Scores ranging from 10 to 17 indicated moderate cognitive impairment. A MOCA score below 10 indicated severe cognitive impairment [67]. The median MOCA score for the participants was 24 (interquartile range: 22–26). Only 35.3% of participants scored within the normal range, while the majority, 57.8%, fell into the mild cognitive impairment group. A smaller proportion, 6.9%, showed moderate cognitive impairment, and no participants exhibited severe impairment. A significant negative correlation was observed between MOCA scores and age (ρ = −0.283, *p* = 0.002). There was a significant correlation between the final MOCA score and CD4 counts at the time of testing (ρ = 0.296, *p* = 0.002). However, no correlation was found with CD4 counts at HIV-1 diagnosis. No correlation was identified between MOCA scores and initial HIV-1 RNA load (ρ = 0.02, *p* = 0.984) or DNA loads.

The MOCA score (<25) was not associated with gender, psychiatric or neurological conditions, type of backbone treatment, or transmission mode. The longitudinal MOCA score changes during the COVID-19 pandemic were determined for 60 PWH who underwent two MOCA evaluations after a median interval of 3.1 years. A slight overall improvement in MOCA scores was observed in the subgroup with repeated testing, with the median score increasing from 24 (interquartile range: 22–26) to 25 (interquartile range: 23–27) (*p* = 0.02). This improvement was particularly noted in recollection, specifically the memory index (*p* = 0.002). Small improvements were also seen in naming and orientation, though these did not reach statistical significance. The proportion of the longitudinal cohort with normal cognitive performance increased from 33.3% to 46.7%. As a result, there were corresponding decreases in the mild (from 61.7% to 50%) and moderate (5% to 3.3%) impairment groups. No participant in the longitudinal cohort had severe impairment at either MOCA evaluation. The study did not find any statistically significant differences in MOCA scores between participants with a history of COVID-19 and those without. This suggests that COVID-19 did not harm cognition in the studied population. Investigators inferred that the MOCA scale helps detect early changes in cognitive functions in PWH and feel the need for additional studies to completely assess the impact of COVID-19 [66].

Most studies focused on post-COVID-19 complications and neurological manifestations did not concentrate on PWH. However, HIV-1 infection status was sometimes included as a co-morbid condition. One such study focused on neurological characterization and biomarker analyses. The analysis included 24 individuals recovering from COVID-19, with 16 participants reporting no neurological symptoms and 8 participants experiencing neurological symptoms. One participant was reported to be HIV-1 positive, four had diabetes, five had hypertension, and five participants had lung disease, which included conditions such as asthma, chronic obstructive pulmonary disease, emphysema, or bronchitis. All participants with neurological symptoms had at least one comorbid condition, while only 43.8% of the participants without neurological symptoms had any comorbid condition. The neurological complaints included general complaints like anosmia, ageusia, altered consciousness, headache, seizures, and paresthesias (abnormal sensations). Participants also reported memory and cognition symptoms. One individual reported double vision, and another reported hallucination. The researchers concluded that there is ongoing neuroinflammation based on several key findings from their analysis of plasma and neuronal-enriched extracellular vesicles (nEVs) in individuals recovering from COVID-19. The neuronal origin of the EVs was confirmed by the presence of synaptophysin. Plasma cytokine interleukin-4 (IL-4) was significantly elevated in all COVID-19 participants compared to controls. Although not significantly increased overall, IL-6 levels showed a trend toward being higher in the group of COVID-19 patients with neurological symptoms compared to those without symptoms and controls. Increased IL-6 was also correlated with age and the severity of sequelae in patients with neurological symptoms. An increase in neurodegenerative protein markers was also observed in the nEVs. Protein markers indicative of neuronal dysfunction, including amyloid beta, neurofilament light, neurogranin, total tau, and one form of phosphorylated tau (p-T181-tau), were all significantly increased in nEVs from all COVID-19 participants compared to controls. In the group experiencing neurological symptoms, neurofilament light and neurogranin significantly correlated with p-T181-tau, suggesting a link between these markers and neurological symptoms. The presence of elevated inflammatory cytokines (IL-4, trending IL-6) and increased levels of neurodegenerative protein markers within neuronal-enriched extracellular vesicles, alongside gene ontology analysis pointing to neuronal damage pathways, collectively led the researchers to conclude that there is ongoing peripheral and neuroinflammation following COVID-19 infection. The authors emphasize the necessity for longitudinal studies to monitor plasma biomarkers and nEV cargo to better understand persistent neurodegeneration and systemic effects following COVID-19 [68]. It would be interesting to see the inclusion of a greater number of PWH in future studies to determine unique post-COVID-19 neurological sequelae.

## 7. Long-COVID in PWH

Long-COVID or PASC is a complex illness affecting multiple systems. It is marked by symptoms lasting at least 12 weeks after the initial COVID-19 infection. A significant number of people who recover from acute COVID-19 experience it, with estimates indicating about 10% may develop long COVID. However, this number is probably underestimated because of under-reporting. The condition can cause serious organ damage and significantly impair function. It also has a major impact on individuals, healthcare systems, and economies [121,122]. Long-COVID symptoms are diverse and can affect multiple organ systems, including the respiratory, cardiovascular, and neurological systems. Common symptoms include fatigue, dyspnea, cognitive dysfunction, chest pain, and myalgia [123]. Pulmonary symptoms such as dyspnea, cough, and wheeziness are prominent in about 6% of cases, particularly in those who had severe acute COVID-19. The pathophysiology of Long-COVID is not fully understood, but it is believed to involve multiple phenotypes driven by different molecular pathways [122]. Diagnosis is primarily based on patient-reported symptoms, as there is no consensus on specific biomarkers or diagnostic criteria [124]. While Long-COVID presents a significant challenge, ongoing research into its mechanisms and management strategies offers hope for more effective, personalized treatments in the future. However, the lack of standardized diagnostic criteria and treatment guidelines remains a critical issue that needs addressing to improve patient outcomes globally.

Long-COVID presents a significant concern for PWH, as they may experience heightened vulnerability due to immune dysfunction and chronic inflammation. Research indicates that approximately 52% of PWH who contract SARS-CoV-2 may develop at least one long COVID symptom, with common manifestations including cough, fatigue, and asthenia [69]. The severity of the initial COVID-19 illness and increased interferon-gamma-induced protein 10 or tumor necrosis factor-α, and decreased interferon-β, among other inflammatory markers, have been identified as risk factors for developing long COVID in the PWH population [69]. Various demographic and clinical factors contribute to the vulnerability of PWH, necessitating awareness among HIV-1 providers regarding potential new or worsening symptoms [41,70].

Long-COVID has a significantly elevated prevalence in PWH. Peluso et al. analyzed 39 PWH and 43 PWoH after COVID-19, using a combined clinical and immunologic approach [125]. They found that HIV-1 status was strongly associated with Long-COVID, with an odds ratio of 4.01 (P = 0.008), and noted distinctive profiles in inflammatory and immune markers [125]. Additionally, a systematic review and meta-analysis encompassing 39,405 participants across 17 studies reported that 52% of PWH with prior SARS-CoV-2 infection experienced at least one long COVID symptom, and HIV-1 more than doubled the odds (OR 2.20, 95% CI 1.25–3.86) [69]. Moreover, RECOVER Program data analyzing over 4 million individuals showed that PWH had a modestly increased risk for long COVID based on phenotype definitions (aOR 1.09–1.18), though associations based on diagnosis codes were less consistent [126].

Neuropsychiatric sequelae of Long-COVID represent a major public health challenge, driven by complex interactions between persistent neuroinflammation, immune dysregulation, and vascular and neuroendocrine dysfunction. Cognitive impairment, anxiety, depression, and sleep disturbances affect up to 40–60% of patients, significantly reducing quality of life and functional capacity. Current evidence supports a multidisciplinary management strategy combining structured neuropsychological assessment, cognitive rehabilitation, and targeted pharmacotherapy, such as SSRIs for mood disorders and stimulants for severe cognitive dysfunction, alongside non-pharmacologic interventions including CBT, mindfulness-based stress reduction, and graded exercise. Adjunctive approaches addressing autonomic dysfunction, nutritional deficiencies, and gut–brain axis alterations may further optimize outcomes. Future research should prioritize biomarker discovery and randomized trials to refine personalized treatment algorithms for this heterogeneous and disabling condition [127].

## 8. Future Directions

The combination of HIV-1 and SARS-CoV-2 infection in the brain may have long-term consequences for neurological function, including cognitive impairment, encephalopathy, stroke, and seizures. In individuals co-infected with both viruses, the risk and severity of these complications may be increased due to synergistic effects on neuroinflammation, cerebrovascular function, and neuronal damage. While almost all the studies we came across do not describe the HIV-1 status, the potential synergy presents an open-ended question worthy of additional examination. PWH may have higher rates of comorbidities such as cardiovascular disease, diabetes, and hypertension, which are risk factors for severe COVID-19 illness and neurological complications. The presence of these comorbidities could further exacerbate the synergistic effects of HIV-1 and SARS-CoV-2 on brain health. Hence, examination of the synergy in HIV-1, especially neuroHIV and COVID-19 cohorts, might be insightful.

Prevention and treatment strategies for PWH largely mirror those recommended for the general population, but thresholds for initiation and the intensity of follow-up should be lower in those with advanced HIV-1 disease (low CD4, high viral load), given the higher complication risk. Vaccination and prompt ART are central. Some data suggest attenuated humoral responses among PWH with low CD4 counts and signals of increased breakthrough infection, supporting optimized booster schedules and early therapeutics [79].

Overall, the potential interplay between these two viral infections in the human brain represents a significant area of concern and requires further investigation to understand the underlying mechanisms and identify potential therapeutic interventions to mitigate neurological complications in individuals co-infected with both viruses. Based on the summation of these studies, we recommend action items in research, clinical, and public policy domains (Table 3).

## Figures and Tables

**Table 1:** Key differences between NeuroHIV and COVID-19 in the human brain. Features compared include nature of infection, primary CNS impact, clinical manifestations, entry mechanisms, and treatment approaches. NeuroHIV is characterized by chronic infection with latent reservoirs in macrophages/microglia, persistent neuroinflammation, and HIV-associated neurocognitive disorders (HAND). COVID-19 primarily involves acute infection, systemic inflammation, microvascular injury, and cognitive symptoms associated with long COVID. Treatment strategies differ: NeuroHIV management relies on lifelong antiretroviral therapy (ART) and adjunctive neuroprotective approaches, whereas COVID-19 management includes antivirals, corticosteroids, symptomatic care for long COVID, and vaccination.

Feature	NeuroHIV	COVID-19
**Nature of Infection**	Chronic, lifelong latent reservoirs in macrophages/microglia	Acute infection, systemic inflammation
**Primary CNS Impact**	Persistent neuroinflammation, synaptodendritic pruning, β-amyloid deposition	Microvascular injury, immune-mediated damage
**Clinical Manifestation**	HAND (HIV-associated neurocognitive disorders)	Cognitive symptoms in long COVID (brain fog, memory issues)
**Entry Mechanism**	Trojan horse via infected leukocytes crossing BBB	Possible direct invasion, systemic cytokine storm
**Treatment Approaches**	Lifelong ART Adjunctive anti-inflammatory/neuroprotective strategies Cognitive rehabilitation	Antiviral agents (e.g., remdesivir) Corticosteroids Symptomatic management for long COVID Vaccination

**Table 2:** Summary of Key Studies on SARS-CoV-2 and HIV-1 Interactions in the CNS and Related Outcomes. Major studies are discussed in the manuscript. They are represented in this table by thematic areas, highlighting their primary focus, key discoveries, and corresponding references. It covers research on COVID-19 incidence and outcomes in people with HIV (PWH), vaccine responses, neuropathology, CNS infection mechanisms, neuropsychological impacts, and long COVID. The table provides a quick reference for understanding the breadth of evidence and knowledge gaps in the interplay between SARS-CoV-2 and HIV-1 within the human brain.

Major Area	Study Focus	Key Discovery	References
SARS-CoV-2 Infection of CNS Cells	In vitro and organoid studies	Astrocytes are primary targets; NRP1 mediates infection; infection induces inflammation and neuronal dysfunction.	Malik et al., 2023 [17]; Yan et al., 2024 [15]; Kong et al., 2022 [18]; Crunfli et al., 2022 [19]; Haverty et al., 2024 [20]; Kettunen et al., 2023 [21]; Proust et al., 2023 [22].
	CNS entry routes	Multiple entry routes: olfactory epithelium, blood-CSF barrier; mixed evidence for olfactory bulb infection.	Studle et al., 2023 [23]; Solar et al., 2025 [24]; Jagst et al., 2024 [25]; Wang et al., 2024 [25]; Shimizu et al., 2024 [26]; Dell’Aquila et al., 2024 [27]; Zhang et al., 2021 [28].
	Autopsy detection of virus	SARS-CoV-2 RNA and protein detected in hypothalamus, cerebellum, spinal cord; replication-competent virus recovered.	Stein et al., 2022 [29]
COVID-19 Incidence & Outcomes in PWH	Impact of pandemic on HIV prevention (PrEP) and COVID-19 risk	COVID-19 disrupted PrEP access globally; increased HIV risk but COVID incidence similar to general population.	Goodreau et al., 2023 [30]; Hong, 2023 [31]; Morgan et al., 2022 [32]; Samra et al., 2024 [33]; Gao et al., 2022 [34]; Kamadjou et al., 2024 [35]; Huang et al., 2022 [36].
	COVID-19 severity in PWH	Low CD4 count and uncontrolled HIV independently increased risk of severe COVID-19 outcomes.	Miller & Gandhi, 2023 [37]; Moller et al., 2023 [38]; Wit et al., 2023 [39]; Nguyen et al., 2024 [40]; Braunstein et al., 2023 [41]
Asymptomatic SARS-CoV-2 in PWH	REPRIEVE cohort and Wuhan study	High asymptomatic infection rates in PWH; morbidity like HIV-negative individuals.	Overton et al., 2022 [42]; Wu et al., 2022 [43]
COVID-19 Vaccine Responses in PWH	Immunogenicity and efficacy studies	PWH show reduced vaccine responses, especially with low CD4 counts; mRNA vaccines perform better; long-term immunity wanes faster.	Wang et al., 2024 [44]; Griffin et al., 2023 [45]; Jongkees et al., 2024 [46]; Zhang et al., 2023 [47]; Benet et al., 2022 [48]; Tuan et al., 2022 [49].
COVID-19 Neuropathology	Autopsy-based studies	Microglial activation, neurovascular injury, fibrin-driven thromboinflammation linked to cognitive deficits; HIV status rarely reported.	Khaba et al., 2020 [50]; Stein et al., 2023 [51]; Shergill et al., 2025 [52]; Fekete et al., 2025 [53]; Lee et al., 2022 [54]; Ryu et al., 2024 [55]
	Post-COVID immune mapping	Dysregulated innate immune response in neuro-Long-COVID brains without overt neuronal damage.	Schwabenland et al., 2024 [56]
	MRI-based mid-term brain changes	Mild COVID-19 can cause microstructural white matter alterations even without hospitalization.	Pelizzari et al., 2022 [57]
Neuropsychological Impact in PWH	Mental health during pandemic	Increased depression, anxiety, and loneliness in PWH; linked to social and structural factors.	Abate et al., 2021 [58]; Delle Donne et al., 2021 [59]; Dyer et al., 2021 [60]; Jones et al., 2021 [61]; Marbaniang et al., 2020 [62]; Matsumoto et al., 2022 [63]; Vindegaard & Benros, 2020 [64]; Hong et al., 2023 [65]
	Cognitive function study	Majority of virally suppressed PWH had mild cognitive impairment; slight improvement over time; COVID-19 did not worsen cognition.	Basoulis et al., 2025 [66]; Nasreddine et al., 2005 [67]
	Biomarker study in post-COVID neuro cases	Elevated inflammatory cytokines and neurodegenerative markers in neuronal EVs; ongoing neuroinflammation post-COVID.	Sun et al., 2021 [68]
Long COVID in PWH	Systematic review and risk factors	~52% of PWH develop long COVID symptoms; severity linked to inflammatory markers and initial illness severity.	Yang et al., 2025 [69]; Martin-Iguacel et al., 2024 [70]

**Table 3:** Actionable Items Checklist for Research and Clinical Strategies on HIV-1 and SARS-CoV-2 Neuroinvolvement: This infographic summarizes key actionable items derived from the review on the synergy between HIV-1 and SARS-CoV-2 infections in the human brain. It organizes recommendations into three major domains: Research Priorities, Clinical & Public Health Actions, and Policy & Infrastructure. Each section highlights critical steps such as investigating neuroinflammatory mechanisms, ensuring uninterrupted HIV prevention services during pandemics, monitoring vaccine responses in People with HIV (PWH), and developing global guidelines for managing co-infections.

Category	Action Items
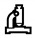 **Research Priorities**	Investigate synergistic effects of HIV-1 and SARS-CoV-2 on neuroinflammation, cerebrovascular function, and neuronal damage in co-infected individuals. Expand studies on ACE2-independent entry mechanisms (e.g., TMEM106B, NRP1) in CNS cells. Conduct longitudinal studies on cognitive outcomes in People with HIV (PWH) post-COVID-19; identify biomarkers. Explore fibrinogen-driven thrombo-inflammation and its role in long-term neurological symptoms. Develop standardized diagnostic criteria and biomarkers for Long COVID, especially in PWH.
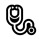 **Clinical & Public Health Actions**	Ensure uninterrupted access to HIV prevention services (PrEP, ART) during pandemics. Screen PWH for cognitive impairment using tools like MOCA; integrate neurocognitive monitoring into HIV care. Monitor vaccine responses in PWH, especially those with low CD4 counts; consider boosters or alternative platforms. Educate HIV care providers about Long COVID symptoms and risk factors in PWH for early intervention.
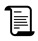 **Policy & Infrastructure**	Include PWH in COVID-19 vaccine trials, perform subgroup analyses for efficacy and safety. Develop integrated mental health support programs for PWH during pandemics. Create global guidelines for managing HIV-1 and SARS-CoV-2 co-infections with emphasis on neurological health.

## Data Availability

No new data were created or analyzed in this study. Data sharing is not applicable to this article.
